# Comment on “Relationship between platelet indices and red cell distribution width and short-term mortality in traumatic brain injury with 30-day mortality”

**DOI:** 10.1590/1806-9282.20231543

**Published:** 2024-05-03

**Authors:** Changjiu Tang, Rong Huang

**Affiliations:** 1 Jingmen People's Hospital, Department of Neurosurgery – Jingmen, China.; 2 Jingmen People's Hospital, Department of Rehabilitation and Physiotherapy – Jingmen, China.

Dear Editor,

Palabiyik et al.^1^ explored the relationship between platelet indices, red cell distribution width (RDW), and short-term mortality in patients with traumatic brain injury. The study aimed to uncover potential associations among these factors, employing a comprehensive analysis with a substantial sample size to bolster the study's reliability. Results indicated no significant differences in mean platelet volume and platelet distribution width values between survivors and non-survivors. Although the platelet count-to-total lymphocyte count ratio values were lower in those who did not survive, this discrepancy did not reach statistical significance. However, within the first 30 days post-traumatic brain injury, deceased patients displayed a notable increase in RDW compared to their living counterparts. Notably, the inclusion of RDW as a parameter in the analysis brought a fresh perspective to the investigation. These findings provide important insights into specific prognostic markers for short-term mortality in patients with traumatic brain injury, emphasizing the significance of considering the RDW levels in the clinical assessment of patients with traumatic brain injury over a 30-day period. However, we note that some concerns require further clarification and elaboration.

First, traumatic brain injury is a diverse condition that may result in significant corticosteroid insufficiency linked to life-threatening illness, contributing to elevated rates of mortality and morbidity. Thus, the clinical management of traumatic brain injury has historically involved the extensive use of glucocorticoids. A meta-analysis^2^, after combining data from 16 studies, shows an increased likelihood of death in patients with traumatic brain injury who were administered substantial doses of glucocorticoids for a brief duration compared to those who received smaller doses over a prolonged period, wherein a tendency toward clinical improvement was noted. Additionally, the application of stress doses of glucocorticoids was associated with a further reduction in pneumonia incidence among patients with traumatic brain injury grappling with critical corticosteroid insufficiency related to life-threatening illness (CIRCI). Evidently, this study^1^ encompasses individuals with traumatic brain injury, a cohort potentially subjected to glucocorticoid exposure. Should these patients indeed have encountered glucocorticoids, the potential repercussions on platelet indices, and red cell distribution width, particularly the platelet count-to-total lymphocyte count ratio, poses a concern. Such an influence could introduce a notable bias in the conclusions drawn from this study. In addition, the information regarding the continuation of glucocorticoid usage post-admission is equally crucial. Hence, it is recommended to furnish a comprehensive account of whether the enrolled patients have utilized glucocorticoids, a measure that would markedly mitigate confounding biases in the analysis and interpretation of the study findings.

Second, it is evident that the major outcomes of this study are the 7- or 30-day mortality rates in patients with traumatic brain injury. However, it is crucial to emphasize that the short-term mortality in patients with traumatic brain injury is not solely linked to platelet indices and red cell distribution width, but it is also influenced by numerous other factors, such as early tracheostomy, the occurrence of acute lung injury (ALI), and acute respiratory distress syndrome (ARDS). Research by Dunham et al.^3^ suggests that, for patients with severe brain injuries, early tracheostomy may not reduce the incidence of ventilator-associated pneumonia, but it can decrease the duration of mechanical ventilation. Another study by Rincon et al.^4^ shows that during a 20-year study period, the prevalence of ARDS/ALI increased from 2% in 1988 to 22% in 2008, and it was associated with a higher risk of in-hospital mortality. However, this present study^1^ only describes mean platelet volume, platelet distribution width, platelet count-to-total lymphocyte count ratio, or RDW, neglecting other factors significantly associated with 7- or 30-day mortality. Therefore, it is imperative to further incorporate factors related to short-term mortality and make additional adjustments in the logistic regression model to accurately identify risk factors closely associated with short-term mortality in patients with traumatic brain injury.
